# Detection of and Early Genomic Insights into Chikungunya Virus, Bolivia, 2025

**DOI:** 10.3201/eid3207.260540

**Published:** 2026-07

**Authors:** Joel Alejandro Chuquimia Valdez, Natalia R. Guimarães, Vagner Fonseca, Cleidy Orellana Mendoza, Sebastián Sasías Martínez, Sara Cândida F. Santos, Gilson Carlos Soares, Mariela Martínez Gómez, Leticia Franco, Lionel Gresh, Jairo Méndez-Rico, Luiz Carlos J. Alcantara, Marta Giovanetti, Leidy Roxana Loayza Mafayle

**Affiliations:** Centro Nacional de Enfermedades Tropicales, Santa Cruz de la Sierra, Bolivia (J.A.C. Valdez, C.O. Mendoza, S.S. Martinez, L.R. Loayza Mafayle); Fundação Ezequiel Dias, Belo Horizonte, Brazil (N.R. Guimarães, S.C.F. Santos); Instituto René Rachou, Belo Horizonte (N.R. Guimarães, S.C.F. Santos, L.C.J. Alcantara); Universidade do Estado da Bahia, Salvador, Brazil (V. Fonseca); Stellenbosch University, Stellenbosch, South Africa (V. Fonseca); Universidade Federal de Minas Gerais, Belo Horizonte (S.C.F. Santos, G.C. Soares); Pan American Health Organization, Washington, DC, USA (M. Martínez Gómez, L. Franco, L. Gresh, J. Méndez-Rico); Università Campus Bio-Medico di Roma, Rome, Italy (M. Giovanetti); Instituto Oswaldo Cruz, Rio de Janeiro, Brazil (M. Giovanetti).

**Keywords:** Chikungunya, CHIKV, arboviruses, viruses, genomic surveillance, molecular epidemiology, transmission dynamics, Bolivia

## Abstract

We report the detection and genomic characterization of chikungunya virus, an arbovirus, during a 2025 outbreak in Bolivia. We identified the circulating chikungunya virus lineage and the transmission dynamics by using genomic surveillance and phylogenetic analyses. Our findings highlight the utility of sustained genomic surveillance for monitoring emerging arboviruses.

Chikungunya virus (CHIKV) is a positive-sense RNA virus belonging to the genus *Alphavirus* (family Togaviridae), primarily transmitted by *Aedes aegypti* and *A. albopictus* mosquitoes. CHIKV is comprised of 3 major lineages: West African, Asian, and East/Central/South African (ECSA). The Asian lineage was introduced into the Americas in 2013, and the ECSA lineage was introduced in 2014. Those introductions gave rise to the Asian-American and ECSA-American sublineages (*1*). Chikungunya infection is typically characterized by acute febrile illness with polyarthralgia, although severe manifestations, including neurologic complications, can occur (*1*). Globally, CHIKV has expanded greatly, with an estimated 16.9 million cases annually and >5.6 billion persons living in at-risk areas (*1*). The Asian-American lineage was first detected in Bolivia in 2015, followed by outbreaks in 2016 and 2017 (*2*). In 2025, a major CHIKV outbreak took place in Bolivia after several years without any reported cases. That outbreak included 4,696 confirmed cases, and most cases (90.8%) were in Santa Cruz (*3*). This resurgence highlights the vulnerability of previously affected regions to new CHIKV outbreaks and underscores the need for sustained surveillance. 

This work is part of the routine arbovirus genomic surveillance implemented in Bolivia. Samples used in this study were obtained anonymously from material exceeding routine arbovirus diagnostics within Bolivia’s public health laboratory network. To investigate the origin and transmission dynamics of the 2025 outbreak, we implemented genomic surveillance of CHIKV in Bolivia. We selected 78 quantitative reverse transcription PCR–positive samples (cycle threshold [Ct] <30), collected from February–June 2025 from 4 departments (Chuquisaca, Cochabamba, Santa Cruz, and Tarija) for our analysis on the basis of Ct value and available metadata (Figure 1). 

**Figure 1:**
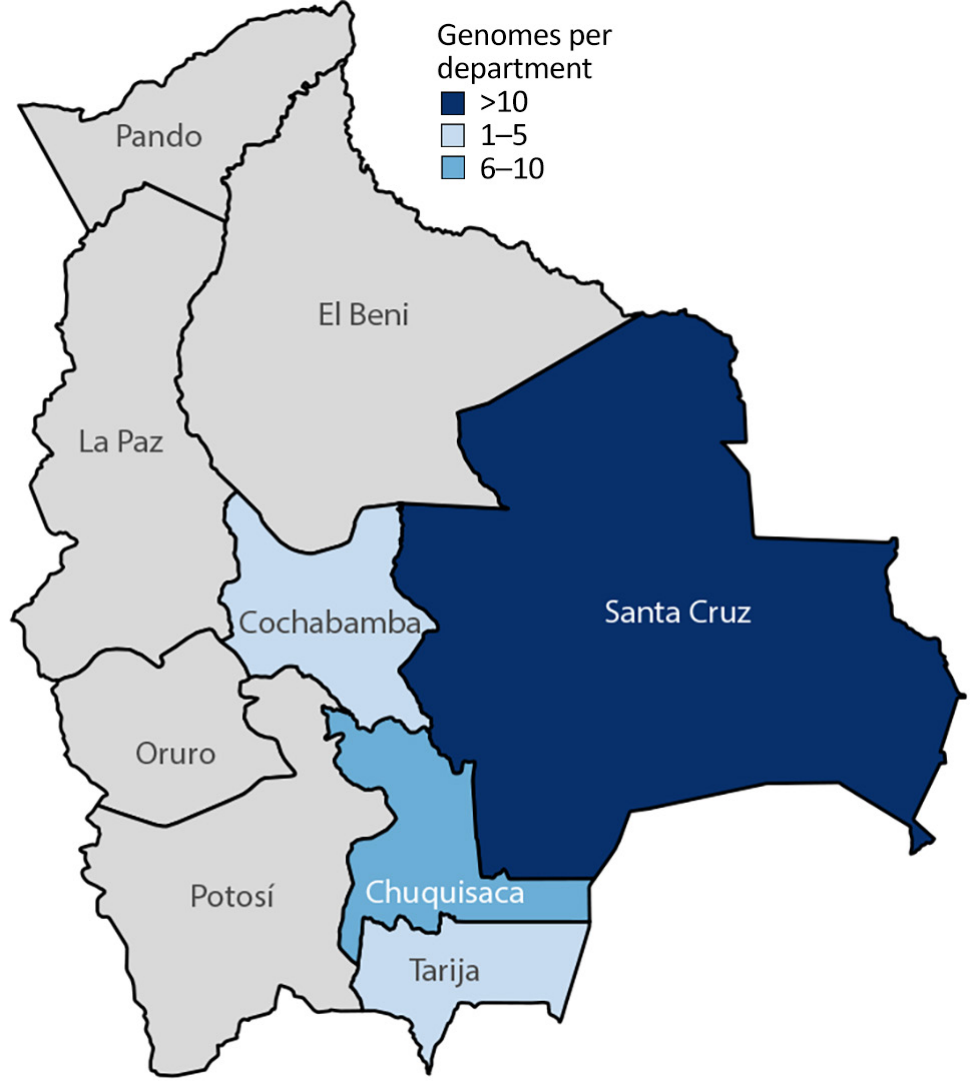
Geographic distribution of sequenced chikungunya virus genomes in a study of chikungunya virus in Bolivia, 2025. Colors indicate the numeric range of genomes per department.

We used a multiplex PCR approach to amplify CHIKV RNA (*4*), and we sequenced CHIKV by using Illumina (Illumina, https://www.illumina.com) and Oxford Nanopore (Oxford Nanopore, https://nanoporetech.com) platforms. We generated consensus genomes by using combined de novo and reference-based approaches (*5*). We conducted a phylogenetic analysis by using genomes from the 78 selected samples together with the 972 publicly available ECSA sequences from the National Center for Biotechnology Information database, which included complete sequences, sampling date, and geographic origin. We performed multiple sequence alignment by using MAFFT (*6*), and we inferred maximum-likelihood phylogenies by using IQ-TREE (*7*). We assessed temporal signal by using root-to-tip regression, yielding a correlation coefficient of 0.52, consistent with sufficient temporal structure for molecular clock inference (*8*). We performed time-scaled phylogeographic reconstruction by using BEAST (*9*) under a relaxed molecular clock model, with an estimated mean evolutionary rate of 2.18 × 10^−3^ substitutions/site/year.

Samples were collected from patients 0–90 years of age, with the highest proportion of samples from patients 0–9 years of age (19.2%, n = 15), followed by samples from patients 20–29 years of age (16.7%, n = 13), and 30–39 years of age (16.7%, n = 13). Most (52.6%, n = 41) samples were from female patients (Appendix 1 Figure 1; Appendix 2 Table). Most patients had acute febrile illness (n = 60), and 18 cases were classified as severe, including 1 encephalitis case and 1 fatal outcome (Appendix 1 Figure 2). 

The sequenced samples had Ct values ranging from 10 to 28 (mean 18.5) (Appendix 1 Figure 3). Sequencing generated 78 near-complete CHIKV genomes with an average genome coverage of 95.9%. All genomes were classified as the ECSA lineage. All CHIKV genomes from Bolivia formed a well-defined monophyletic clade, with the closest ancestry linked to viruses circulating in Midwest Brazil (Figure 2, panel A). This genomic analysis suggests that the 2025 CHIKV outbreak in Bolivia was driven by a single introduction event followed by sustained local transmission. Time-scaled phylogeographic analysis estimated CHIKV introduction around November 2024 (95% CI late October–early November). The earliest transmission was inferred in Chuquisaca, followed by dissemination to Santa Cruz and subsequent spread to Tarija and Cochabamba (Figure 2, panel B). The inferred directional spread toward more densely populated regions further supports the role of human mobility and urban transmission networks in enabling rapid geographic expansion (*10*). 

**Figure 2:**
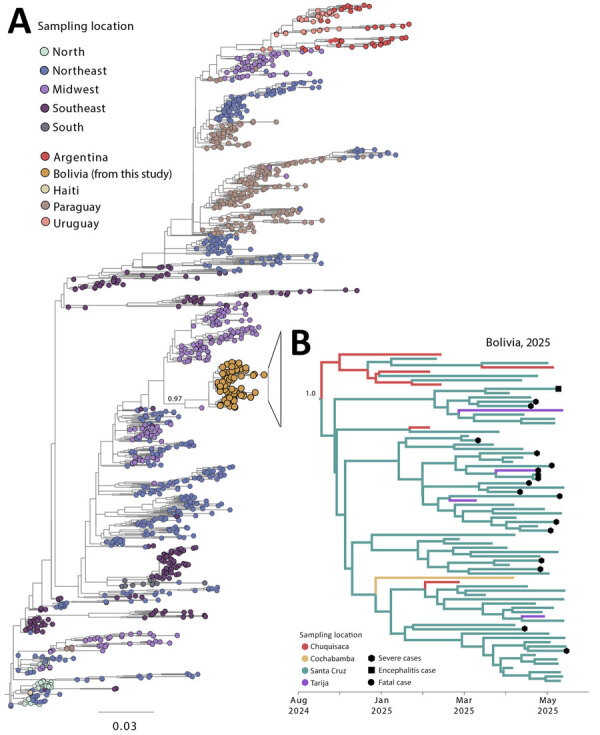
Regional genetic diversity and temporal spread of the chikungunya virus outbreak in Bolivia within the Americas, 2025. A) Phylogenetic tree showing the placement of genomes from Bolivia within the broader diversity across the Americas. Tips are colored according to sampling origin, and sequences from Bolivia are highlighted. The genomes from Bolivia cluster in a well-supported group, consistent with local expansion. B) Time-scaled tree of the Bolivian clade illustrating temporal progression and geographic distribution across departments (Chuquisaca, Cochabamba, Santa Cruz, and Tarija). Symbols indicate severe cases (including 1 encephalitis case and 1 fatal case). Scale bar indicates nucleotide substitutions per site.

Our results reveal the genetic similarity of CHIKV strains circulating in Bolivia during the 2025 outbreak and provides evidence indicating a single introduction of CHIKV from Midwest Brazil, with subsequent spread across multiple departments. Our findings improve our knowledge of CHIKV transmission dynamics in Bolivia; however, limitations in temporal and geographic sampling coverage might have limited full characterization of viral diversity. Our findings also demonstrate how integrating genomic surveillance into outbreak investigations enables identification of introduction events and reconstruction of transmission pathways, providing critical insights to inform public health interventions. Because of increasing arboviral activity across the Americas, those approaches are essential to improve early detection and guide timely response strategies.

Appendix 1 Additional information and figures about detection and early genomic insights into chikungunya virus, Bolivia, 2025.

Appendix 2 Epidemiological and sequencing details of chikungunya virus samples from Santa Cruz de la Sierra, Bolivia.

## References

[1] de Souza WM, Lecuit M, Weaver SC. Chikungunya virus and other emerging arthritogenic alphaviruses. Nat Rev Microbiol. 2025;23:585–601. 10.1038/s41579-025-01177-8 40335675

[2] França CMB, Loayza R, Roca Y, Montaño Arias AM, Tinajeros F, Loaiza JR, et al. Genome sequences of chikungunya virus isolates from Bolivia. Microbiol Resour Announc. 2020;9:e00230–20. 10.1128/MRA.00230-20 32299879 PMC7163017

[3] Pan American Health Organization. Epidemiological alert: chikungunya. 2026 [cited 2026 Mar 27]. https://www.paho.org/en/documents/epidemiological-alert-chikungunya-10-february-2026

[4] Quick J, Grubaugh ND, Pullan ST, Claro IM, Smith AD, Gangavarapu K, et al. Multiplex PCR method for MinION and Illumina sequencing of Zika and other virus genomes directly from clinical samples. Nat Protoc. 2017;12:1261–76. 10.1038/nprot.2017.066 28538739 PMC5902022

[5] Vilsker M, Moosa Y, Nooij S, Fonseca V, Ghysens Y, Dumon K, et al. Genome Detective: an automated system for virus identification from high-throughput sequencing data. Bioinformatics. 2019;35:871–3. 10.1093/bioinformatics/bty695 30124794 PMC6524403

[6] Katoh K, Standley DM, Yamada KD. MAFFT multiple sequence alignment software version 7: improvements in performance and usability. Mol Biol Evol. 2013;30:772–80. 10.1093/molbev/mst010 23329690 PMC3603318

[7] Nguyen LT, Schmidt HA, von Haeseler A, Minh BQ. IQ-TREE: a fast and effective stochastic algorithm for estimating maximum-likelihood phylogenies. Mol Biol Evol. 2015;32:268–74. 10.1093/molbev/msu300 25371430 PMC4271533

[8] Rambaut A, Lam TT, Max Carvalho L, Pybus OG. Exploring the temporal structure of heterochronous sequences using TempEst (formerly Path-O-Gen). Virus Evol. 2016;2:vew007. 10.1093/ve/vew007 27774300 PMC4989882

[9] Baele G, Ji X, Hassler GW, McCrone JT, Shao Y, Zhang Z, et al. BEAST X for Bayesian phylogenetic, phylogeographic and phylodynamic inference. Nat Methods. 2025;22:1653–6. 10.1038/s41592-025-02751-x 40624354 PMC12328226

[10] Chadsuthi S, Althouse BM, Iamsirithaworn S, Triampo W, Grantz KH, Cummings DAT. Travel distance and human movement predict paths of emergence and spatial spread of chikungunya in Thailand. Epidemiol Infect. 2018;146:1654–62. 10.1017/S0950268818001917 29983134 PMC9507951

