# Supplementary material for: Detection of and Early Genomic Insights into Chikungunya Virus, Bolivia, 2025
Source: Emerg Infect Dis. 2026 Jul;32(7):1198–201. doi: 10.3201/eid3207.260540 (PMC13322436; doi:10.3201/eid3207.260540)
Supplement: Appendix 1 — Additional information and figures about detection and early genomic insights into chikungunya virus, Bolivia, 2025. [file 26-0540-Techapp-s1.pdf]

Article DOI: https://doi.org/10.3201/eid3207.260540

EID cannot ensure accessibility for supplementary materials supplied by authors. Readers who have difficulty accessing supplementary content should contact the authors for assistance.

Detection of and Early Genomic Insights into Chikungunya Virus, Bolivia, 2025

Appendix 1

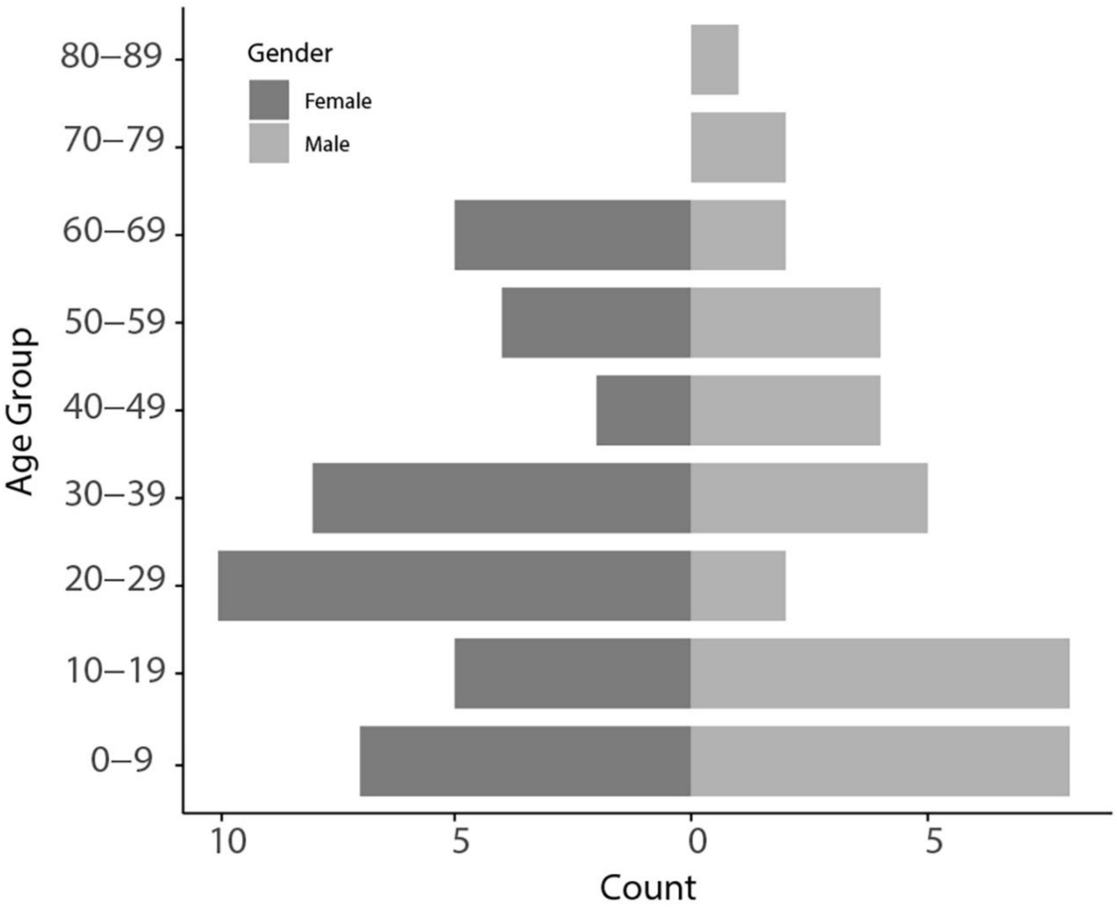

Appendix 1 Figure 1. Age in years and sex distribution of the study population. Bars represent counts within each age group.

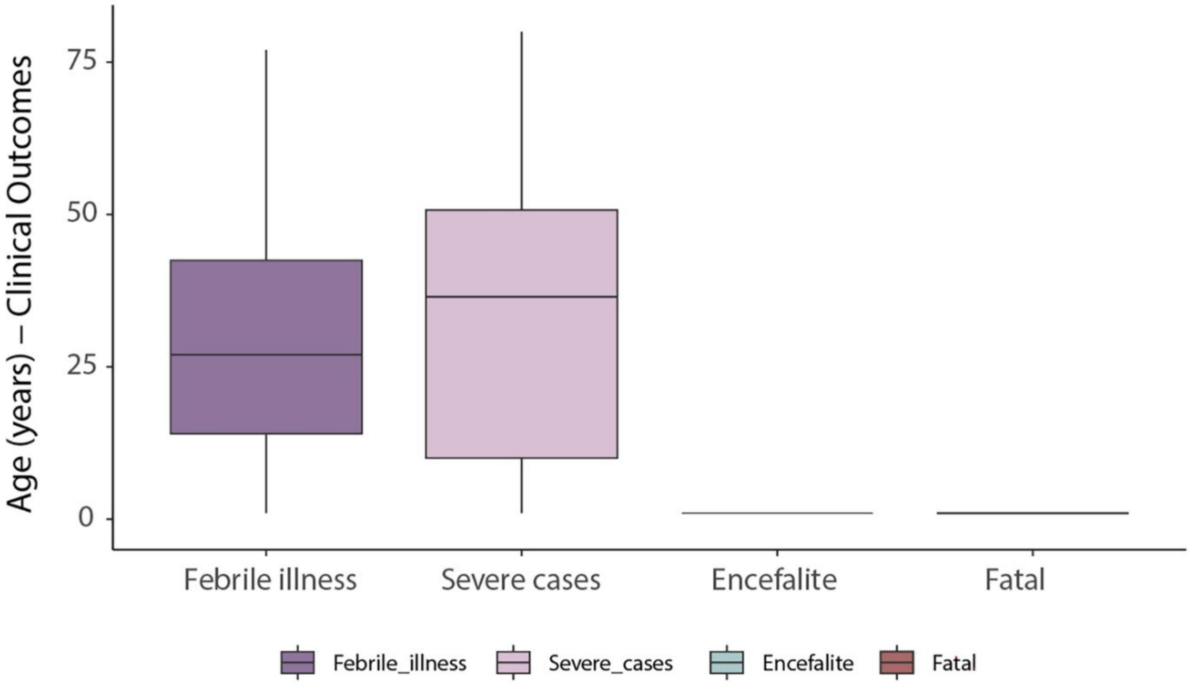

Appendix 1 Figure 2. Age in years distribution according to clinical outcome (febrile illness, severe cases, encephalitis, and fatal cases). Boxplots display the median, interquartile range, and range.

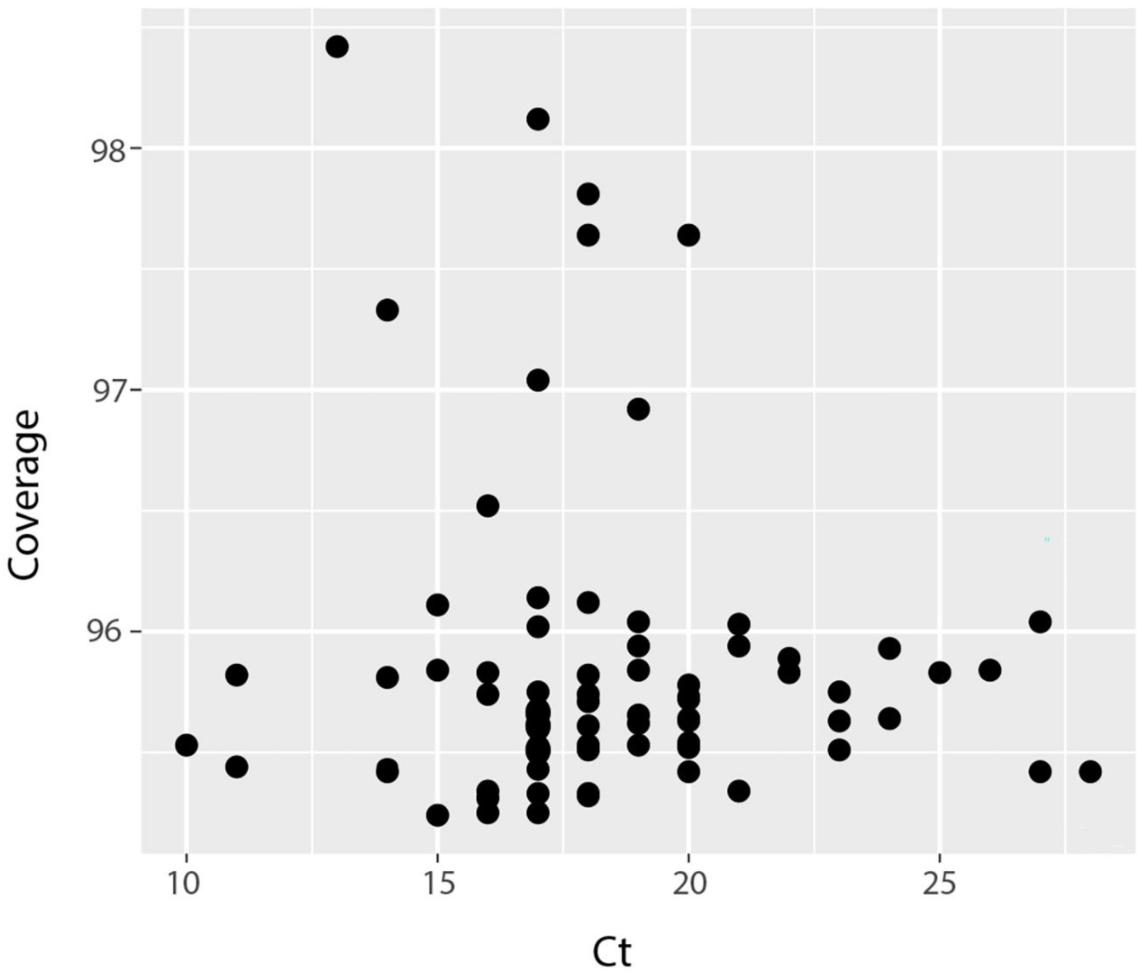

Appendix 1 Figure 3. Relationship between cycle threshold values and genome coverage. Each point represents an individual sample.
